# Cohort profile: The Czech Health, Alcohol and Psychosocial factors In Eastern Europe study (HAPIEE-CZ)

**DOI:** 10.21203/rs.3.rs-8261173/v1

**Published:** 2026-04-09

**Authors:** A. Dalecká, A. Peasey, Y. Pan, N. Čapková, L. Šebejová, P. Piler, M. Kozela, A. Pajak, A. Tamosiunas, L. Andrýsková, J. Klánová, M. Marmot, H. Pikhart, M. Bobák

**Affiliations:** 1 RECETOX, Faculty of Science, Masaryk University, Brno, Czech Republic; 2 Research Department of Epidemiology and Public Health, University College London, London, UK.; 3 National Institute of Public Health, Prague, Czech Republic.; 4 Department of Epidemiology and Population Studies, Institute of Public Health, Jagiellonian University Medical College, Krakow, Poland.; 5 Laboratory of Population Research, Institute of Cardiology, Lithuanian University of Health Sciences, Kaunas, Lithuania.; 6 Institute of Health Equity, University College London, London, UK.

**Keywords:** Cohort study, Longitudinal design, Epidemiology, Biomarkers, Ageing, Mortality

## Abstract

**Background:**

The HAPIEE (Health, Alcohol and Psychosocial factors In Eastern Europe) is a population-based longitudinal prospective cohort study established to explore psychosocial, behavioural, and environmental risk factors of chronic conditions and ageing outcomes, including their trajectories, in middle aged and older urban populations.

**Methods:**

Four representative urban cohorts of middle-aged men and women were established in the Czech Republic (n = 8,856), Poland (n = 10,727), Lithuania (n = 10,940), and Russia (n = 9,363) in the early 2000s. This cohort profile update summarises the main design features across the four cohorts, followed by the recent re-examination of the Czech cohort in 2023–2024. At this most recent data collection, Czech participants aged 64–90 years completed postal questionnaire (n=2,350), performed physical and cognitive tests (n=1,519) and provided blood samples (n=1,388).

**Findings to date:**

The HAPIEE study is the largest multi-country cohort established in Eastern Europe, incorporating harmonized longitudinal data from four populations. The HAPIEE data have led to a considerable scientific contribution, including over 100 published papers in peer-reviewed journals. Large absolute and relative all-cause mortality inequalities in Eastern Europe were explained by measures of socio-economic position and material deprivation, including food access and living conditions. Additionally, increased frailty index, increased depressive symptoms, and poor cognitive functions were found as significant risk factors of all-cause mortality. The HAPIEE cohort contributed to development and validation of the ESC SCORE2 risk assessment model.

**Future directions:**

Data from all four cohorts were linked to national administrative registers offering a comprehensive view of the participants’ health status over more than 20 years. Additionally, geocoding of the participants’ addresses opened the opportunities to link data to numerous external exposome characteristics and census data. Lastly, DNA samples of Czech participants who provided blood sample at baseline underwent whole genome genotyping allowing investigation of the contribution of genetic predispositions (DNA polymorphisms, polygenic scores) to the various health conditions.

## Background

The HAPIEE (Health, Alcohol and Psychosocial factors In Eastern Europe) prospective cohort study was established in Central and Eastern Europe (CEE) in the early 2000s to provide extensive evidence on psychosocial and socio-economic determinants of cardiovascular and other non-communicable diseases (NCDs) [1]. The original research questions focused on explaining the high cardiovascular morbidity and mortality observed in the former communist countries during the 1970s and 1980s, resulting in a large gap in life expectancy between eastern and western Europe [2,3]. The main hypotheses focused on the role of heavy and binge drinking, low consumption of fruits and vegetables and psychosocial distress on cardiovascular diseases as they had been suggested as key risk factors of both increased risk and inequalities within countries and for the gap in mortality between eastern and western European countries [4,5].

In addition, the rapid increase in life expectancy observed in post-communist countries during the 1990s encouraged growing interest in healthy ageing among CEE populations [5]. Despite this improvement, healthy life expectancy remains shorter in CEE compared to Western Europe [6], posing challenges for the health system, economy and social policy. In consequence, the research focus of the HAPIEE study, whose oldest participants reached 90 years of age in 2023, was expanded to investigate the causes and consequences of declines in physical, mental and cognitive abilities, frailty, multi-morbidity and well-being [1].

Prospective cohort studies, recruiting large population-representative samples, have shown to be a highly effective and efficient approach to reveal the causes of diseases and understand the trajectories of the aging-related outcomes over the repeated measurements. Thus, the HAPIEE study focused on the key domains capturing the changes in health status and their risk determinants, while maintaining the comparability of measurements with previous data collections (e.g. healthy behaviours, quality of life, psychosocial factors including social networks, social trust and perceived control over life, depression symptoms, physical functioning, cognitive functioning).

## Cohort description

### Original study design

The HAPIEE cohort was designed as a prospective study, including four representative urban cohorts of middle-aged (45–69 years) men and women established in the Czech Republic (n = 8,856), Poland (n = 10,727), Lithuania (n = 10,940), and Russia (n = 9,363). Random samples, stratified by gender and 5-year age groups were selected from population registers. The baseline investigation was conducted in 2002–2005 in the Czech Republic, Poland and Russia; the Lithuanian cohort joined the project in 2006–2008. Data were collected by structured questionnaires and health examination, including blood sampling. The baseline formal consent included permission for linkage with mortality registers in all countries, allowing regular updates of date and cause of death of deceased participants. Participants of wave 1 in the Czech Republic, Poland and Russia were reinvited during 2006–2008 for the second wave of data collection (this was the baseline in Lithuania). Since then, the follow-up differed between the cohorts (Figure S1).

The study has been coordinated by Department of Epidemiology and Public health at UCL, UK. Each cohort has been managed by local principal investigators, including the National Institute of Public Health, Prague and RECETOX, Masaryk University, Brno (since 2023) in the Czech Republic; the Department of Epidemiology and Population Studies, Jagiellonian University Medical College, Krakow, in Poland; the Department of Population Studies, Institute of Cardiology of Kaunas University of Medicine in Lithuania; and the Institute of Internal Medicine, Russian Academy of Medical Sciences, Novosibirsk in Russia [1].

**Fig. 1:**
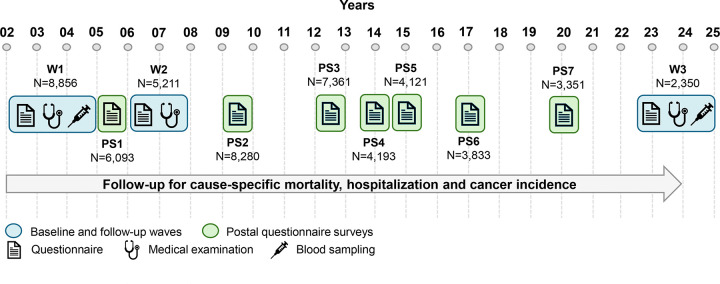
Timeline of data collection in the HAPIEE-CZ study

This particular cohort profile focuses on the most recent re-examination (Wave 3) of the Czech cohort (HAPIEE-CZ), because a) the Polish follow-up examination began shortly before the COVID-19 pandemic lockdown (2021), during which the funding was discontinued, and this ultimately made its continuation impossible (only 464 participants were examined, and 2,076 completed a postal survey); and b) collaboration with the Russian cohort was suspended due to the Russian invasion of Ukraine. The timeline of the Czech data collection is presented in Figure 1.

### Follow-up examination of the HAPIEE-CZ

As mentioned above, Czech participants were followed up by postal surveys (PS) every 2–3 years (PS1 – PS7), with relatively high response rates (70–80% among eligible subjects). This most recent data collection that was conducted in the participating seven Czech cities (Hradec Králové, Liberec, Ústí and Labem, Jihlava, Kroměříž, Karviná, Havířov) in 2023–2024 (Wave 3).

**Fig. 2:**
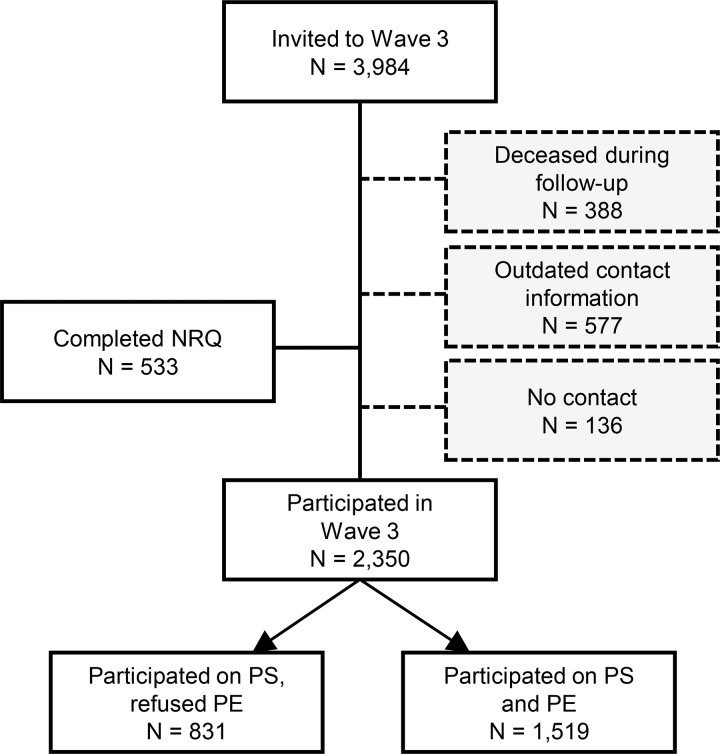
Flow diagram of the HAPIEE-CZ re-examination (Wave 3) in 2023–2024s Note: PS – postal questionnaire survey; PE – physical examination; NRQ – Non-respondent questionnaire

From the original sample of 8,856 participants, 2,744 individuals died, and 2,128 had unknown or outdated contacts or requested to withdraw from the study before the invitation to the follow-up examination (before 1^st^ March, 2023), leaving 3,984 participants who were eligible for the follow-up examination. The questionnaires together with invitation letter for physical examination were sent by post to all eligible participants. In cases of no response, participants from whom a phone number was known from previous data collections were approached by phone at interval of 2–3 weeks after initial invitation. After a positive response, participants were contacted by phone to make an appointment for a physical examination (PE). In total 2,350 individuals participated in follow-up examination, although the participation rates of the postal questionnaire survey and physical examination differed as not all participants were willing or able to participate in person on examination (Figure 2).

### What has been measured?

The follow-up examination consisted of postal questionnaire survey (PS), physical examination (PE), and collection of a venous blood sample.

#### Questionnaire.

The postal survey aimed to collect data on health, physical functioning, health behaviours and health literacy, quality of life, psychosocial factors, community-level characteristics, occupational risks, economics, social status, and residential history. A detailed overview of measured variables throughout data collections is provided in Table 1.

#### Physical examination.

The examinations were conducted in the healthcare facilities by trained nurses. It included measurement of anthropometric characteristics (weight, height, sitting height, waist and hip circumferences), blood pressure, spirometry, walk speed, grip strength and cognitive testing (memory, concentration and verbal skills). Prior to blood pressure measurement, participants were asked to sit quietly for 5 minutes. Blood pressure was measured three times, with a two-minute interval between measurements, using an Omron M5-I digital blood pressure monitor. Lung function was assessed with Spirosonic Smart spirometer (using SpiroReporter software to store curves in electronic format). Prior to spirometry measurement, participants were asked for contraindications (myocardial infarct or eye operation in 3 months before the examination). The spirometry manoeuvre was conducted at least three times followed by the quality control according to the ERS/ATS 2017 standards [7]. Lung function parameters included forced vital capacity (FVC), forced expiratory volume in 1 second (FEV1), peak expiratory flow (PEF). The predicted values were calculated according to the GLI 2012 reference equations, accounting for age, sex, height and ethnicity of the participants [8]. Grip strength was measured on both hands using Scandidact Smedley’s dynamometer [9]. Participants were asked for contraindications (arm surgery in 6 months before the examination, recent injury accompanying swelling or pain) prior to the measurements. Four cognitive domains, namely immediate and delayed memory, verbal fluency, and processing speed, were tested using standard tests, including word recall, animal naming, and letter cancellation [10]. First, a list of 10 words was recorded and played to participants over three consecutive and one delayed 1-min trials. Second, the participant was asked to name as many different animals as possible within 1 min. Third, the letter cancellation task instructed participants to cross out the letters “P” and “W” from a grid of randomly chosen letters as accurately as possible within 1 min (range 0–65).

#### Blood sampling.

Participants were not required to be fasting prior to blood sampling. The samples (n=1,388) were collected using one S-Monovette^®^ Serum Gel tube (1 × 7.5 ml) and two S-Monovette^®^ EDTA K3E tubes (1 × 7.5 ml and 1× 2.7 ml). The collected blood samples were processed within one hour of collection. The S-Monovette^®^ Serum Gel tube (7.5 ml) and S-Monovette^®^ EDTA K3E tube (7.5 ml) were centrifuged at 2500g for 10 minutes. The resulting fractions (plasma, serum, buffy coat) were separated into new sterile test tubes. The S-Monovette^®^ EDTA K3E tube (2.7 ml) was homogenized and whole blood was manually aliquoted into three cryotubes (700 μl per aliquot). All processed blood samples/fractions were stored at −20 °C in the local healthcare facilities and regularly transferred to CELSPAC biobank, RECETOX, Masaryk University. The plasma and serum fractions were thawed overnight at 4°C, aliquoted automatically (250 μl per aliquot) by Hamilton Microlab STAR. The aliquotes in FluidX cryotubes are stored in the Askion automated cryogenic storage module at −150°C. Whole blood and buffy coat fractions are stored in ultra-low temperature freezers at −80°C.

#### Biomarkers.

An extensive set of biomarkers have been currently analysed, including 1) *cardiometabolic* (total cholesterol, high-density lipoprotein, low-density lipoprotein, total triglycerides, glucose, glycated haemoglobin); 2) *renal* (blood urea nitrogen, uric acid, creatinine, cystatin C); 3) *inflammatory* (C-reactive protein, rheumatoid factor); 4) *hepatic* (gamma-glutamyl transferase, alanine aminotransferase, aspartate aminotransferase, alkaline phosphatase); 5) *endocrine* (dehydroepiandrosterone sulphate, vitamin D, thyroid-stimulating hormone); 6) *bone metabolism* (osteocalcin); and 7) *cell damage and muscle function* (creatine kinase, creatine kinase-MB, lactate dehydrogenase, alpha-amylase, N-terminal pro b-type natriuretic peptide, albumin) biomarkers.

#### Mortality, hospitalization and oncological follow-up.

The Czech mortality data has been updated quarterly since 2002; the latest update was provided in December 2024. The Czech linkage also included Czech National Oncological Register (last update in “02/2024”) and the Czech National Register of Hospitalizations (last update in “12/2024”), enabling investigation of onset and progression of diseases. Thus, the register data provide a comprehensive view of the participants’ health status over more than 20 years (Figure 1).

#### GWAS.

In early 2024, DNA samples of participants who provided blood sample at baseline underwent whole genome genotyping using the Illumina GSA v3 array. Quality control has been completed, and the data are being currently processed.

Data entry of the questionnaires and physical examination (Wave 3) was done using EpiData Manager software, version 4.6.0.6 (EpiData Association, Denmark). A full sample of forms was double entered for quality assurance. Table 1 summarizes the data availability in the HAPIEE-CZ study.

### Characteristics of the HAPIEE-CZ population

Table 2 shows demographic, health behaviour and physical functioning characteristics of three groups of the individuals participated in the most recent re-examination (Wave 3), including a) individuals who participated in both PS and PE (n=1,519); b) individuals who participated in PS only (n=831); and c) individuals who completed only non-respondent questionnaire (NRQ) (n=533). In brief, the individuals who participated on both PS & PE are younger, subjectively healthier, reporting substantially less limitations in climbing stairs, walking and shopping compared to individuals who participated only in PS or who completed NRQ. There are no substantial differences in hearing and healthy behaviours, specifically smoking status between the group of PS & PE and PS only (Table 2). Additionally, those who participated on both PS & PE in W3 were younger, more educated and were less physically limited at baseline compared to those who decided not to participate in W3 (Table S1).

### Areas of future research

The study collected extensive data on ageing characteristics, including cognitive abilities, grip strength, walk speed, activities of daily living (ADL), instrumental activities of daily living (IADL) that were measured repeatedly. Thus, the HAPIEE-CZ cohort is well suited for longitudinal analysis enabling investigation of trajectories of ageing-related outcomes [11] and their biological, genetic and epigenetic biomarkers reflecting gradual changes in functional status.

The second new direction of study relates to area-based exposures. The Czech cohort participants’ home addresses have been geocoded. This allowed linkage with census data at the level of small settlement units (n=320 in HAPIEE-CZ; 2021), providing a more nuanced understanding of socio-economic disparities affecting specific population groups. Data from three census measurements (2001, 2011 and 2021) are available.

Third, the geocoding of the participants’ addresses opened the opportunities to link to numerous external exposome (environmental) characteristics, including air pollution, noise, green spaces, blue spaces, temperature, light at night. The HAPIEE-CZ study has been recently incorporated into several European research partnerships focusing on understanding the complex interactions between environmental exposures, health behaviours (e.g. diet, physical activity, smoking) and human health [12]. The exposome framework also helps to elucidate how social determinants of health influence individual’s likelihood of being in at risk from harmful environmental exposures [13].

Finally, we recently completed whole genome genotyping of the full HAPIEE-CZ cohort. This will allow investigation of the contribution of genetic predispositions (DNA polymorphisms, polygenic scores) to the various health conditions (e.g. cardiometabolic diseases or cancer). Moreover, the integration of genetic data with longitudinal information on environmental exposures enables the exploration of gene-environment interactions, offering deeper insights into how genetic susceptibility modulates the impact of environmental risk factors.

### Key findings to date

The HAPIEE data have been widely used in diverse discipline, and have led to a considerable scientific contribution, including over 100 published papers in peer-reviewed journals. Below, we provide several highlights of the publications that have contributed to the epidemiological understandings of various health outcomes, including mortality, morbidity, physical functioning and cognitive aging.

### Mortality

- Increased frailty index [14], increased depressive symptoms [15], and poor cognitive functions [16] were found as significant risk factors of all-cause mortality.
- Large absolute and relative all-cause mortality inequalities in Eastern Europe were explained by measures of socio-economic position and material deprivation, including food access and living conditions [17].
- High adherence to the traditional Eastern European dietary pattern was linked with increased risk of all-cause and cardiovascular mortality [18], and poor cognitive functioning [19].
- The HAPIEE cohort contributed to development and validation of the ESC SCORE2 risk assessment model [20].

### Physical functioning, ageing and morbidity

- Obesity was associated with less favourable physical functioning at baseline and a faster decline over 10 years in Russian males and females [21].
- Accumulation of disadvantaged socio-economic position over the life course was associated with impaired lung function, mainly driven by disadvantages in young and late adulthood [22].
- A substantially higher proportion of underdiagnosed and poorly treated cases of diabetes and hypertension was observed in Eastern compared to Western European countries [23].

### Health-behaviour and environmental risk factors

- Around one third of cancer deaths in males may have been attributable to smoking and/or alcohol consumption in Eastern Europe [24].
- A combination of *FTO* and *ADH1B* polymorphisms with smoking status appeared to influence the risk of binge drinking in females [25].
- Living in air polluted, less green and socio-economically deprived areas was associated with worse cognitive functioning in Czech population, partially explained by physical activity [26].

### Strengths and weaknesses

To our knowledge, the HAPIEE study is the largest multi-country cohort established in Eastern Europe, incorporating harmonized longitudinal data from four populations. The main strength of the HAPIEE cohort is its longitudinal study design, with repeated measures on a wide range of multidisciplinary topics, providing evidence on health and ageing in Eastern Europe. The study benefits from strong participant retention over more than 23 years, which makes it possible to study slow-developing diseases such as neurodegenerative diseases or specific types of cancer. A further strength is the record linkage with routinely collected data from health registers, which offers valuable addition to data collection. Data linkage also provides a rich and continuous source of data of environmental and socio-economic factors influencing health and ageing. Moreover, the four HAPIEE studies are well-harmonized, following the same study protocol that enables cross-country comparisons. Finally, the most recent follow-up examination created new research opportunities to explore underlying mechanisms in numerous associations, using newly collected biomarkers and genetic data.

Several weaknesses need to be acknowledged. First, more than 30% of the Czech original study participants have already died, and around further 35% of survivors did not participate in the most recent follow-up, reducing the representativeness of the re-examination study sample. Thus, not all study findings may be generalizable to broad ageing population as on average healthier individuals with better physical functioning participated in Wave 3. Additionally, the repeated postal surveys provide self-reported data, which may not always be unbiased.

## Supplementary Material

This is a list of supplementary files associated with this preprint. Click to download.

• Supplementaryfile.docx

## Figures and Tables

**Table 1:** Overview of questionnaire and physical examination data available in the HAPIEE study

Domain	Variables	02–05	05–06	06–08	09	12	13–14	15–16	17–18	20–21	23–24
		W1	PS1	W2	PS2	PS3	PS4	PS5	PS6	PS7	W3
		Q/PE	Q	Q/PE	Q	Q	Q	Q	Q	Q	Q/PE
Health	Medical history in last 12 months	✔	-	✔	✔	✔	✔	-	✔	✔	✔
	Current medication	✔	-	✔	-	-	-	-	-	-	-
	Self-rated health	✔	-	✔	✔	✔	✔	✔	✔	✔	✔
	Injuries in last year	✔	-	✔	-	-	-	-	-	-	✔
	Awareness & treatment of high BP, high cholesterol and diabetes	✔	-	✔	✔	✔	✔	✔	✔	✔	✔
	Detailed history of myocardial infarction and stroke in last 12 months	✔	✔	✔	-	✔	✔	✔	✔	✔	✔
	Cough	✔	-	-	-	-	-	-	-	-	✔
	Depression CESD-10 (CESD-20 at W1)	✔	-	✔	✔	✔	✔	✔	✔	✔	✔
	Dental health	-	-	-	-	-	-	-	-	-	✔
	Hearing & vision limitations	-	-	-	-	-	✔	-	-	-	✔
	COVID-19 infection	-	-	-	-	-	-	-	-	-	✔
	Menopause & hormonal therapy	✔	-	✔	✔	-	-	-	-	-	✔
	Sexual life	-	-	-	-	-	-	-	-	-	✔
	Measured anthropometry characteristics	✔	-	✔	-	-	-	-	-	-	✔
	Self-reported height and weight	✔	-	-	-	-	-	✔	✔	✔	✔
	Blood pressure & heart rate	✔	-	-	-	-	-	-	-	-	✔
	Pulmonary functions	✔	-	-	-	-	-	-	-	-	✔
	Expectations of future health and economic situation	-	-	✔	-	-	-	-	-	-	-
Physical functioning	Physical functioning (from SF36)	✔	-	✔	✔	✔	✔	✔	✔	✔	✔
	ADL & IADL	-	-	✔	-	✔	✔	✔	✔	✔	✔
	Frailty	-	-	-	-	-	-	-	✔	✔	-
	Walk speed	-	-	✔	-	-	-	-	-	-	✔
	Chair rise	-	-	✔	-	-	-	-	-	-	-
	Grip strength	-	-	✔	-	-	-	-	-	-	✔
Cognitive functioning	Memory	✔	-	✔	-	-	-	-	-	-	✔
	Concentration	✔	-	✔	-	-	-	-	-	-	✔
	Verbal fluency	✔	-	✔	-	-	-	-	-	-	✔
Health behaviours	Sleep	✔	-	-	-	✔	✔	-	-	-	✔
	Smoking	✔	-	✔	✔	✔	✔	✔	✔	✔	✔
	Alcohol, pattern & problem drinking	✔	-	✔	-	-	-	-	-	-	✔
	Food frequency	✔	-	-	-	-	-	-	✔	✔	-
	Physical activity	✔	-	✔	-	-	-	-	✔	✔	✔
	Health literacy	-	-	-	-	-	-	-	-	-	✔
Quality of life	CASP-19 (retired only)	✔	-	-	-	-	-	-	-	-	-
	CASP-12 (all participants)	-	-	✔	-	-	-	✔	-	✔	✔
Psychosocial factors	Social networks	✔	-	✔	-	✔	✔	✔	✔	✔	✔
	Perceived control over life	✔	-	✔	-	-	-	-	-	-	-
	Social participation	✔	-	✔	-	-	-	-	-	-	✔
	Caring for sick/disabled person	-	-	-	-	✔	✔	✔	✔	✔	-
	Loneliness	-	-	-	-	-	-	✔	✔	✔	✔
Community-level characteristics	Social trust	✔	-	✔	-	-	-	-	-	-	✔
	Collective efficacy & Perception of reciprocity	-	-	✔	-	-	-	-	-	-	✔
Occupational risks	Occupational risks (noise, dust, chemicals, physical demands etc.)	-	-	-	-	-	-	-	-	-	✔
	Job control/demand (working only)	✔	-	-	-	-	-	-	-	-	-
	Effect/reward imbalance (working only)	✔	-	✔	-	-	-	-	-	-	-
Economics	Current economic activity	✔	✔	✔	✔	✔	✔	✔	✔	✔	✔
	Housing tenure	-	-	✔	-	-	-	-	-	-	✔
	Household amenities	✔	-	✔	-	-	-	-	-	-	✔
	Benefits	✔	-	✔	-	-	-	-	-	-	✔
	Material difficulties	✔	-	✔	-	✔	✔	-	✔	-	✔
	Unemployment history	✔	-	✔	-	-	-	-	-	-	✔
	Housing conditions/cohabitation	✔	-	✔	-	✔	✔	✔	✔	✔	✔
	Retirement & reason of early retirement	✔	-	✔	-	-	-	-	-	-	✔
	COVID-19 pandemic consequences	-	-	-	-	-	-	-	-	✔	✔
Social status	Education	✔	-	✔	-	-	-	-	-	-	-
	Marital status	✔	✔	✔	✔	✔	✔	✔	✔	✔	✔
	Childhood social circumstances	✔	-	✔	-	✔	-	-	-	-	-
	Employment history	✔	-	✔	-	-	-	-	-	-	✔
Residential history	Residential history since aged 35	-	-	-	-	-	-	-	-	-	✔

PS – postal survey; Q – Questionnaire; PE – physical examination

**Table 2:** Demographic, health-related and lifestyle characteristics of the HAPIEE-CZ participants and non-respondents in Wave 3 (2023–2024)

	PS & PE	PS only	NRQ
Total	1,519	831	533
***Demographic characteristics***			
Age (mean, SD)	75.2 (6.4)	77.0 (6.4)	79.0 (6.5)
Male, %	39.1	38.3	37.9
Female, %	60.9	61.7	62.1
Single, %	2.2	1.3	-
Married/cohabiting, %	59.3	58.3	-
Divorced, %	14.6	10.9	-
Widowed, %	23.9	29.5	-
***General health and limitations***			
Poor/very poor self-rated health, %	8.7	16.1	35.0
Very limited when climbing several flights of stairs, %	14.5	25.8	35.7
Very limited when walking 1 km, %	10.6	19.4	37.2
Very limited when shopping, %	3.2	8.7	24.0
Poor/very poor hearing, %	10.4	11.6	-
***Lifestyle characteristics***			
Current smokers, %	10.0	11.9	-
Alcohol consumption at least once a week, %	39.7	32.5	-

PS – postal survey; PE – physical examination; NRQ – non-respondent questionnaire

## Data Availability

The Czech data are available via request with a description of proposed research questions and hypotheses from the Department of Epidemiology and Public Health, University College London, UK (e-mail: h.pikhart@ucl.ac.uk or m.bobak@ucl.ac.uk) and RECETOX, Masaryk University, Czechia (e-mail: andrea.dalecka@recetox.muni.cz). A submitted application will be reviewed within a few weeks, followed by signing the MDTA (Material and/or Data Transfer Agreement). Because the HAPIEE study is a not-for-profit cohort, we do not charge any costs for data preparation and release. More information together with a study protocol can be found at https://www.ucl.ac.uk/epidemiology-health-care/hapiee-study. Files with syntax enabling replication of the results are available at: https://zenodo.org/uploads/17790255.
